# Public commentary on teacher quality: an analysis of media comment on the teaching performance assessment

**DOI:** 10.1007/s13384-023-00635-7

**Published:** 2023-05-23

**Authors:** Donna Pendergast, Beryl Exley, Frances Hoyte

**Affiliations:** School of Education and Professional Studies, Griffith University, Mount Gravatt, QLD Australia

**Keywords:** Teaching performance assessment, Initial teacher education, Accreditation, Legacy media, Social media, Teacher quality

## Abstract

In Australia, the Teaching Performance Assessment (TPA) is a relatively new, mandatory hurdle which must be completed just prior to the graduation stage of initial teacher education (ITE) programmes. This high-stakes task is one of a growing number of requirements to come out of the standards and accountability regime as outlined in the Australian Institute for Teaching and School Leadership (AITSL) document for accreditation for ITE programmes. We delve into the public commentary about the broader commission of preservice and graduate teacher quality in general and the TPA in particular. We draw on Bernstein’s pedagogic identities and deductively apply this theory to explore this phenomenon. We use a data set of publicly available legacy media and social media tweets made over a ten-month period from August 2019 to May 2020 to reveal the focus, inherent bias and pedagogic identities promoted by these public discourses. The paper concludes with discussion about the implications of these drivers on the public perception of quality in ITE and on the status of teaching more broadly.

## Initial teacher education in context

According to UNESCO, the last few years has evidenced the continual global ‘preoccupation’ with teacher education, recruitment, retention, professional status and working conditions for teachers (UNESCO Institute for Statistics, 2020). There is also an estimated shortfall of 69 million teachers to achieve the universal goal of primary and secondary education by 2030 (UNESCO Institute for Statistics, 2020). Throughout the world, teacher education institutes, the school systems who employ graduate teachers, and the regulatory authorities who accredit teacher education programmes are all drawing on a range of policy, accountability, practice, research and equity ‘turns’ (Cochrane-Smith, 2016; Goodwin, 2020; Zeichner, 2020) to remodel teacher education. The re-imagining of teacher education in Australia is one response to teacher shortages, the effects of which are dramatic, with workforce shortfalls in all school systems in all states and territories of Australia (see Singhal, 2019, April 21). A convergence of factors contributes to teacher shortages, including increased population, a decline in applicants to ITE, and public commentary about the low status of and declining morale within the profession (Commonwealth of Australia, 2019; Exley et al., 2022). Despite the fact that many issues contribute to teacher shortages, initial teacher education has taken the brunt of the blame in policy and public commentary (Rowan et al., 2015; White et al., 2020).

Concurrently, the teaching profession in Australia is also faced with allegations of reduced teacher quality in response to student achievement data. Critics cite plummeting performance in international comparative scales, such as the Programme for International Student Performance (PISA). Australia’s achievement trends in PISA reveal a net downward spiral in all domains over more than two decades (Thomson et al., 2019). The broader commission of teacher quality is thus constantly commented upon. For instance, Carey and Hunter reported in the *Sydney Morning Herald* that ‘Australian students have recorded their worst ever results in an international test of reading, maths and science skills, and are now about a full school year behind where Australian students were at the turn of the millennium’ (2019, December 3, para. 1). Declining student achievement has spearheaded school- level initiatives such as implementing uniform centralised testing regimes across the country’s diverse schooling systems. Declining student achievement has resulted in more calls for accountability and standardisation initiatives to improve teacher quality and ensure ‘classroom ready graduates’. Such initiatives have specifically targeted ITE.

A series of government- initiated inquiries into the profession has resulted in the establishment of policy making government entities designed to shape teacher education and build accountabilities aligned with the teacher quality agenda (Allard et al., 2014; Rowan et al., 2015). The establishment of the Australian Institute for Teaching and School Leadership (AITSL) in 2010 serves as a particular marker, commencing the journey to establish (i) professional standards for teachers; and (ii) standards for ITE programmes. These standards are federally mandated requirements for programme accreditation, with ITE graduates eligible for teacher registration and subsequently employment as a teacher. Since AITSL was established, national level inquiries have continued to focus on quality in ITE and the teaching profession more generally. The highly influential Teacher Education Ministerial Advisory Group’s *Action Now: Classroom Ready Teachers* (TEMAG, 2014) review made 38 key recommendations designed to renovate ITE, respond to concerns about the diminishing status of the profession, and address the attrition of novice teachers (many within 5 years of graduation). In this review, the cause of this attrition is largely framed as inadequate preparation in their ITE programme.

As a result of the advice to TEMAG (AITSL, 2020, Para.1), and with extensive consultation, the Programme Standards document *Accreditation of Initial Teacher Education Programmes in Australia: Standards and Procedures* (AITSL, 2018), was developed in 2011 and revised in 2018, with accreditation overseen by the state-based authority. These standards comprise a suite of mandatory requirements including academic and non-academic selection criteria for programme entry (Programme Standard 3.2), the Literacy and Numeracy Test for Initial Teacher Education (Programme Standard 3.5), English language proficiency requirements (Programme Standard 3.6), and a minimum number of days of professional experience (Programme Standard 5.2). Another mandatory requirement is an exit hurdle task that demonstrates readiness for teaching—the Teaching Performance Assessment (TPA). The establishment of a TPA requirement in Australia follows international trends to replace written licensure tests to document the readiness of graduate teachers to enter the classroom. For example, a version of the TPA was first piloted in California in 2004, and then mandated for newly enrolled preservice teachers from 2008 (Campbell et al., 2016). Since this time, the Californian TPA has been redesigned and renamed as the Fresno Assessment of Student Teachers (FAST), and the Performance Assessment for California Teachers (PACT). Common across all versions is a focus on the assessment of the graduate teacher’s planning, instruction and assessment of their own practices and students’ learning (Campbell et al., 2016).

Our focus in this article is on the public commentary around one specific initiative in ITE, the introduction of a TPA in Australia. In the next section, we introduce the background information about the TPA initiative. We then locate and analyse the mentions of this initiative in publicly available legacy media and social media tweets over a 10-month period. We draw on Bernstein’s (2000) pedagogic identities and deductively explore this phenomenon. Our analysis demonstrates the misunderstanding that exists, and we examine why some stakeholders lack a voice on this matter. We close by commenting on the possible repercussions of the public’s perception of the TPA on ITE and graduate teacher quality.

## Teaching Performance Assessments in Australia

After considerable work to design and implement TPAs in Australia (see e.g., Allard et al., 2014; Kriewaldt et al., 2021), these assessments are now a key component of the mandatory hurdles all ITE students complete to graduate, achieve registration and be eligible for employment. Developed in response to Recommendation 28 of the TEMAG Report, ITE providers and schools are required to ‘assist pre-service teachers to develop and collect sophisticated evidence of their teaching ability and their impact on student learning’ (TEMAG, 2014, p. 33). The inclusion of a TPA in ITE programmes enables providers to meet Programme Standard 1.2 which specifies that ‘Program and assessment design ensures pre-service teachers successfully complete a final-year *teaching performance assessment* prior to graduation’ (AITSL, 2018, p. 12). On their website, AITSL (2022) explains that a TPA is:

a tool used to assess the practical skills and knowledge of pre-service teachers. Pre-service teachers collect evidence of practice to complete a TPA in the final year of their initial teacher education programme. It is assessed by ITE providers and is a requirement of graduation. (AITSL, 2022, “What is a teaching performance assessment?” section)

Since their inception, TPAs have continued to come under scrutiny as part of policy reviews of ITE. Attracting and selecting high quality candidates and preparing ITE students to be effective teachers was the focus of the *Quality Initial Teacher Education* (QITE) review (Tudge, 2021). The resulting report, *Next steps: Report of the Quality Initial Teacher Education Review* (Australian Government, 2021) emphasised the value of the TPAs, stating they were:

regarded as the most significant outcome of the TEMAG Review reforms, with the most potential to improve the quality of ITE programmes and produce graduate teachers with the skills, knowledge and practices to be successful when they enter the classroom. (Australian Government, 2021, p. 44)

By the end of 2021 all TPAs utilised in Australian ITE achieved endorsement. An estimated 25,000 preservice teachers attempt one of the 12 approved TPAs each year. The two most widely used are the *Graduate Teacher Performance Assessment* (GTPA) led by the Australian Catholic University, and the *Assessment for Graduate Teaching* (A*f*GT) led by Melbourne University. The GTPA requires ITE students to teach a significant learning sequence with clear learning goals and differentiation for individual learners and small groups. ITE students must demonstrate planning, teaching, assessing, reflecting and appraising practices (Australian Catholic University [ACU], 2018). A validated, quality assured assessment process combines internal and external moderation with common standards across all 18 participating providers. Likewise, the A*f*GT brings together 14 institutions and requires demonstration of planning for learning and teaching; analysing teaching practice; assessing for impact on student learning; and expanding practice. Together, these two TPAs account for the majority of higher education institutions (32 of 47), with the remaining TPAs a combination of small consortia and TPAs produced by individual higher education providers.

As well as emphasising the importance of TPAs, the *Next Steps* report (Australian Government, 2021) identified challenges with the early implementation, concluding that various stakeholders claimed that a lack of consistency in quality was ‘limiting the potential impact of TPAs’ (Australian Government, 2021, p. 44). The report recommended a ‘strengthening’ of TPAs through ‘national setting and moderation and comparability’ (p. 54). The goal of ‘classroom readiness’ was to be achieved through more accountability and tighter control of the range of TPAs, with decision-making power given to a new, external ‘governance board’, limitations on provider attempts to have their TPA endorsed, and more government funding to support the two major TPAs (Australian Government, 2021, p. 54).

The introduction of TPAs is a complex move in the ITE space. On one hand, TPAs can facilitate comparability and accountability imposed on ITE by external forces, supporting what Rowe (2017) describes as the ‘market responsive’ approach, as opposed to the ‘capacity building’ approach favoured in Finland (p. 7). On the other hand, TPAs offer the potential for higher education providers to have input into the design and content of this capstone task. Allard et al. (2014) suggest that ITE providers have had a major role in creating and implementing the task and that this has given them some agency in preparing and assessing graduates (see also Loughland & Bostwick, 2022). Additionally, the major TPA tasks in Australia provide preservice teachers with the type of task that has potential to reflect the complexity of teaching, that is, contextualised problem solving, requiring engagement with theory, reflection, and a repertoire of teaching skills (Reid, 2019).

### Market-oriented thinking

The approach to both ITE and school education in Australia has been described by Rowe (2017) as ‘market-oriented’ (p. 7), that is, relying on standardisation and testing to measure performance. The trend towards standardisation is evident in schooling (Simpson Reeves et al., 2018), and in teacher education via the implementation of high stakes tasks for certification and in government control of accreditation processes for teacher education programmes (Cochrane-Smith et al., 2017; Goodwin, 2020).

Scholes et al. (2017) note that in the Australian context, a strong focus on teacher quality has thwarted a consideration of other factors that may influence student achievement such as socioeconomic backgrounds, ethnicity, refugee status and gender, and students’ geographic location. The overriding power of discourse of teacher quality also pertains to the ITE space, with the recent focus in the TEMAG and QITE reviews on the quality of graduate teachers, and a philosophical commitment to a market-oriented stance demonstrated through the further ‘strengthening’ of elements such as the TPA. The push for standardisation of content in ITE and benchmark testing to measure preservice and graduate teacher performance is the foundation of the new accreditation processes for ITE programmes across the country. As a consequence, ‘many recent reforms target classroom teacher quality and readiness’ (Rowe, 2017, p. 7). These reforms have been enacted in Australia via the accreditation requirements mandated by AITSL, specifically increasing accountability and establishing a range of standards that reinforce market-oriented thinking. Given this market-oriented model driving education policy makers in Australia, we are keen to delve deeper into the public’s understanding of TPAs as arguably the strongest lever in the ITE reforms.

### Public commentary

Our examination of the legacy media (which include, for example, online newspapers, Canter, 2018) and social media comment on the TPA sits against a backdrop of public commentary on preservice and graduate teacher quality in Australia and elsewhere. Comments about the TPA have potential to become part of the broader public conversation about the teaching profession (Exley et al., 2022). Since the digitalisation of media, and the burgeoning access to the Internet, this public commentary on ITE quality has proliferated in disparate ways. Commentary, written by journalists and featured in the legacy media, provides limited opportunity for readers and other stakeholders to challenge dominant discourses, which often drive negative public opinion about ITE quality in Australia (Keogh & Garrick, 2011) and internationally (see Goodwin, 2020). Baroutsis and Lingard (2019) also highlighted the propensity with which the mainstream media in Australia constructs education as being in crisis and successes as being atypical.

While commentary via social media allows individual citizens and stakeholder representatives to engage in a/synchronous banter from which journalists are typically left out (Canter, 2018), other factors have been found to constrain the teaching profession’s engagement in these discussions. Pendergast et al. (2019) analysed three publicly accessible blog posts about teacher professionalism in Australia in 2018. The general public, activist groups and publishing companies joined the discussion about teacher professionalism, but many teachers and school leaders were ‘rendered voiceless by their employers’ in this space. Being active on social media was considered ‘time-consuming, intellectually and emotionally exhausting, and at times, risky business’ (Pendergast et al., 2019, p. 47). This latter point is reinforced by Baroutsis and Woods (2020) who reported that when an Australian teacher educator provided a professional viewpoint about a teaching matter, their @handle was bombarded with unsavoury tweets by those with an opposing viewpoint who hid behind cryptic @handles. In another case, three Australian parents who did not agree with a school’s enactment of a behaviour management process were each found guilty in an Australian court for unfairly savaging a principal’s reputation on social media. This case made international headlines as *Australia’s most epic parent-teacher row EVER* (Wilkie, 2020, March 2). Research by Willis and Exley (2018) found that some teachers had limited experience with social media or lacked the time and technical support to participate. For a number of reasons, teachers often lurked rather than participated, meaning that within the social media mode, the voices of the profession were often mediated by a few (Exley et al., 2022). In light of the public discussion of the profession, graduate teachers and ITE, we were interested to see how both these media channels respond to and represent the TPA, a recent innovation in ITE in Australia.

#### Theoretical framing

To examine Australian public commentary on preservice and graduate teacher quality generally and the TPA specifically, we drew on Bernstein’s (2000) theorisation of discourse. According to Bernstein (2000), discourses external to the higher education provider, such as discourses of the state (Hoyte et al., 2020) or discourses from major stakeholders (Exley & Kitson, 2018), prescribe and proscribe the way ITE programmes are designed, delivered and assessed. The public commentary, via both legacy and social media, is palpable and powerful in that it contributes directly and indirectly to the ebb and flow of what constitutes a quality ITE programme and a valid TPA. These understandings contribute to the shaping of the institutional identity of the ITE programme. Bernstein (2000) proposes that the range of discourses may project, or give prominence to, one or more of four possible institutional identities: retrospective identities, prospective identities, therapeutic identities, and market driven identities.

- A Retrospective Identity (RI) is projected when institutions dis-embed and de-locate criteria from the past and project them into the present programmes (Bernstein, 2000). RI discourses are dominant when, for example, an ITE programme is not modified to meet the needs of the current cohort of preservice and graduate teachers or current market imperatives. In their examination of two teacher education programmes in Australia, O’Meara and MacDonald (2004) asserted that only elite institutions with considerable reputational capital can afford to project a RI and attract academics with a strong commitment to RI who in turn perpetuate RIs. An example of RI discourses in ITE is the high stakes Literacy and Numeracy Test in Initial Teacher Education (LANTITE) which all preservice teachers must pass to graduate.
- A Prospective Identity (PI) is projected when institutions recontextualise criteria from the past alongside a neo-conservative push for social change, such as cultural, economic and technological change (Bernstein, 2000). A PI dominates in ITE programmes when the institution has the ‘desire to foreground the future career needs of teachers through the selective recontextualising of past features’ (O’Meara & MacDonald, 2004, p. 114). An example is the overt placement, within an ITE program, of Australian Professional Standard for Teachers (APST) ‘2.4 Understand and respect Aboriginal and Torres Strait Islander people to promote reconciliation between Indigenous and non-Indigenous Australians’ (AITSL, 2018, p. 22). As Exley and Chan (2014) document, this APST usually requires new content knowledge for many preservice and graduate teachers.
- A Therapeutic Identity (TI) is projected when institutions give prominence to progressive pedagogies that subscribe to complex theories of personal, social and cognitive development (Bernstein, 2000). For example, if the institution builds its reputation on the formal teaching qualifications and years of experience of their academic and sessional staff and advertises a focus on the personal development and actualisation of preservice and graduate teachers as individual autonomous and flexible thinking learners, then the institution would be projecting TIs. Bernstein (2000, p. 70) explains that the power within TI discourses can be disguised by ‘soft management styles and veiled hierarchies’ (2000, p. 69). TIs are expensive because time and effort are required to develop the theoretical basis; theory is not always explicit, and the outputs of the learning are difficult to standardise and thus quantify.
- A Market Identity (MI) is formed when institutions give priority to the local employment market requirements (Bernstein, 2000). When institutions view their work in teacher education as fulfilling a vocational role rather than a knowledge exploration role, a MI dominates. Influential market sources include systems of education and individual schools who supervise and assess preservice teachers and employ graduate teachers. When MI projections dominate, market sectors exert measures to ensure institutional compliance via funding allocations, such as mandating or banning ITE content. When MI projections are most active, ITE programmes are vulnerable to re-modelling in response to current issues, state initiatives and employment market demands (O’Meara & MacDonald, 2004).

While multiple discourses circulate at any one time, one or more may gain prominence to drive public perceptions of preservice and graduate teachers (Rowan et al., 2015) and influence current understandings of the TPA. Bernstein (2000) and others (O’Meara & MacDonald, 2004; Pausigere & Graven, 2013) use the nomenclature of pedagogic identities to refer to the projection of an institutional identity and its mediation by macro, mezzo and micro level discourses. In this way, this work on pedagogic identities sits apart from other research work that explores identity in teacher development (see Beauchamp & Thomas, 2009), and in teachers’ professional lives (see Olsen, 2008).

## The study

We investigated the public discourses about the quality of Australian ITE programmes and the newly introduced TPA over a 10-month period from 1 August 2019 to 31 May 2020. Our research question is: What are the discourses driving the public commentary on AITSL’s Teaching Performance Assessment task in Australia and what are the inherent implications? We investigated both legacy and social media, in recognition of the complexity of the communication landscape (Canter, 2018; Pendergast et al., 2019).

Our data collection period sits between the commencement of TPAs as obligatory components of ITE, and before Stage 2 of Accreditation when institutions would have to start publishing the results of their TPA processes. During the first months of the study, preservice teachers were able to implement the data collection, planning and teaching required to complete their TPA in face-to-face classroom contexts. With the onset of the global COVID-19 pandemic and resulting school closures (Australian Government & Department of Health, 2020), from late March 2020, preservice teachers completed an adapted TPA task, designed collaboratively by ITE providers and education systems (see Pendergast et al., 2020). The data collection period also includes two points in time where graduating preservice teachers were distributing their eportfolios and seeking employment opportunities. This article focuses on commentary on the TPA in both its original format and also the alternative TPA.

### Methods

#### Harvesting data set #1: legacy media

Australian legacy media includes two national newspapers and 10 state/territory daily papers, published in print and online. Just two companies dominate ownership of the national papers, one owned by News Corp Australia (formerly News Limited) and the other by Nine Entertainment Co (formally Fairfax Media). These two companies also own eight of the state/territory daily newspapers.

Articles for data set #1 were identified through searches on two occasions in each of two databases: the Australia New Zealand Newsstream (via the ProQuest platform) and the Australia and New Zealand Reference Centre (via EBSCOhost platform). The first set of searches covered 1 August 2019 to 31 March 2020. The second set of searches covered 1 April 2020 to 31 May 2020. Search terms included the following phrases: ‘teaching performance assessment’ and ‘teacher performance assessment’, the terms ‘TPA’, ‘teacher graduate’, ‘graduate teacher’, ‘graduate teach*’, ‘preservice teachers’, and ‘teacher standards’. This variety of terms was used because the TPA is a relatively new assessment in Australia and it may have been referred to without use of its formal name. The articles located by the searches were read to identify ones about Australia that included comments relevant to the TPA.

The first search in the Australia and New Zealand Newsstream (via Proquest) delivered eight relevant articles. The search in the Australia/New Zealand Reference Centre (via Ebscohost) identified no additional articles. The second round of searches located one reference from the Proquest platform and one additional reference from the Ebscohost platform. A total of one Letter to the Editor and 11 articles comprise the legacy media data set. Five articles were from the Brisbane-based Courier Mail (News Corp), three from the Sydney Morning Herald (Nine/Fairfax Media) and one each from The Cairns Post (News Corp), the Courier (Ballarat) (News Corp), and The Age (Nine/Fairfax Media) published in Melbourne. Articles 11 and 12 were published during the time schools in some Australian states moved to online learning due to COVID restrictions.

#### Harvesting data set #2: social media

Social media posts were located using two search engines: Social Searcher and Google Twitter Search, at three points in time. The first searches covered August 2019 to March 2020 and the second covered April 2020 to June 2020. These searches used the terms ‘teacher performance assessment’, ‘graduate teacher’ and ‘teacher standards’. The Google Twitter search provided four posts and a small trail of responses. The Social Searcher tool provided two Instagram posts and a notification of a private Facebook group discussing the Graduate Teacher Performance Assessment (GTPA). A third search in July 2020 with the search term ‘teaching performance assessment’ located two Instagram posts from Social Searcher and six posts from the Google Twitter Search.

The Instagram posts provided a rich seam of data. Both Instagram posts included a selection of hashtags which linked the post to virtual ‘noticeboards’ of other posts which were potentially on the same topic. Any given hashtag might relate to a number of topics (e.g., #afgta was linked to posts about fitness and the #tpa linked to the United States versions of a teaching performance assessment). We read several of the most promising hashtag notice boards (see Table 1) to locate posts about the Australian TPA from within our timeframe.

**Table 1:** Information on the hashtags with potential relevance for the TPA

Hashtags explored	TPA-related posts
#afgta	3 posts
#tpa	Unrelated topics and TPAs in the US
#graduateAQ teacher	A small number only relevant to Australia
#assessment for graduate teaching	One post- “Miss-Happy” (pseudonym)
#teacher performance assessment	Two posts relevant to Australia
#gtpa	46 posts relevant to Australia
#teaching performance assessment	2 posts relevant to Australia

The hashtag #gtpa led to a set of 46 social media posts (see Table 2). These posts were distributed over the data collection timespan, and many were cross linked to other relevant hashtags which also revealed a small number of posts with similar content and tone. We chose to limit our discussion to the #gtpa posts because this set clear limits to the social media we discuss.

**Table 2:** Number of #gtpa posts per month from August 2019 to June 2020

Month	Aug	Sept	Oct	Nov	Dec	Jan	Feb	Mar*	Apr*	May*
#gtpa posts	5	5	8	1	0	3	5	9	4	6

*Schools in some Australian states moved to online learning from the last week of March 2020 due to COVID restrictions

In summary, the data on which we report includes 12 articles from the legacy media and the 46 social media posts under the #gtpa.

### Analysis of data

To analyse public commentary on the TPA within the Australian ITE context we considered the dominant discourses with respect to the pedagogic identities proposed by Bernstein (2000). The third author conducted the first pass through data sets #1 and #2 to determine the themes that emerged, that being statements about the decline in Australia’s educational standards; the exclusive use of external testing as the measure of educational standards; the inadequacy of teachers; the reliance on government-initiated reviews to drive reforms; the selection criteria for programme entry; and the evaluation of graduates prior to entry to the profession. In a series of face-to-face data analysis meetings, all authors read each legacy and social media article and worked deductively using the definitions and understandings of the pedagogic identity theory to note overt or implicit orientations to particular identities. This allowed us to specify orientations to pedagogic identities for the topic of teacher quality. We came up with the following examples:

- Retrospective identities (RI) orientate to criteria from the past that are projected into the current programme (e.g., comments about applying external standards, the assumed benefits of LANTITE)
- Prospective identities (PI) orientate to criteria from the past aligned to social change (e.g., comments about teacher commitment or bringing about change)
- Therapeutic identities (TI) orientate to pedagogies subscribing to complex theories of personal, social and cognitive development (e.g., comments on empathy, resilience, or creativity)
- Market identities (MI) orientate to the career needs of the local employment market (e.g., references to workforce demands dictating changes to teaching subject areas).

### Findings

#### Analysis data set #1: legacy media

The 12 legacy media articles in data set #1 had various authors: journalists (Articles 1, 7, 8, 10, 11), the Director of the Queensland College of Teachers (Articles 2/3 & 6), a State Government Minister (Article 5), a researcher (Article 4) and a private citizen who wrote a letter to the editor (Article 9). See Table 3 for information about date, publisher, headline, author, summary and identified orientations.

**Table 3:** Analysis of legacy media artefacts

Article, Date, Source & Author	Headline & summary	Orientation
Article 1 1 October 2019 Sydney Morning Herald (2019)	“Teachers could learn from report” Release of report from NSW Auditor General: Impose standards to raise teacher quality	Standards as the means to improve graduate teacher quality (RI)
Article 2/3 25 October 2019 The Courier Mail The Cairns Post Fishburn (2019a, b)	“Being a teacher takes more than you might think.” “Valuing the work of teachers” High standards to enter the profession, including “extensive professional experience, a rigorous degree, & a challenging final teaching performance assessment” Teachers need- extensive skills, deep content knowledge, creativity, intelligence, empathy, adaptability & resilience” (p. 16)	Orientations to standards (RI), teachers as empathetic, adaptable & resilient (TI)
Article 4 4 December 2019 The Courier Mail Joseph (2019)	“Slipping standards to blame for poor result” “mediocre standards” for ITE LANTITE benchmarks for ITE entry ensure rigorous ITE content	Standards as the means to improve graduate teacher quality (RI)
Article 5 7 December 2019 Sydney Morning Herald Mitchell (2019)	“Recognise that marks matter to stop 20-year education slide” PISA results ATAR entry scores “poor university marks”	Standards as the means to improve graduate teacher quality (RI)
Article 6 17 January 2020 The Courier Mail Fishburn (2020)	“Rest assured, Queensland’s teachers are the best” Entry scores,motivation & qualities for teaching such as empathy, adaptability, resilience, passion to make a difference and service orientation TPA and LANTITE	Orientations to standards (RI), passion to make a difference (PI), & empathy, adaptability & resilience (TI)
Article 7 17 January 2020 The Courier Mail O’Flaherty (2020)	“Education standards defended” Entry standards include academic & non-academic capabilities LANTITE & TPA	Orientations to standards (RI), & non-academic capabilities (PI)
Article 8 18 January 2020 The Courier Mail Lang (2020)	“Raise the bar for better teachers” Pick ‘brightest minds’ Entry standards Entry requirements are to address teacher shortage Teachers need “soft skills such as creativity, communication & empathy”	Orientations to standards (RI), workforce needs dominating standards (MI), & need for creativity, communication & empathy (TI)
Article 9 20 January 2020 The Courier Mail MacLean (2020)	“Teaching Passion Columnist” Entry into ITE -suitability for teaching, “passion & ability to communicate” “we should attract the best & brightest”	Orientations to standards (RI), & teachers as having passion & being able to communicate (TI)
Article 10 24 January 2020 The Courier Hopkins (2020)	“FedUni professor secures prestigious scholarship” Scholarship to research TPAs TPAs- relatively new in Australia	No discernible orientation to a particular identity for preservice & graduate teachers
Article 11* 27 April 2020 The Age Carey (2020)	“Teacher trainee class hours slashed to avoid shortfall” No easing of academic standards ATAR for entry LANTITE	Standards as the means to improve graduate teacher quality (RI)
Article 12* 29 May 2020 SMH Baker (2020)	“Calls for national plan to attract top students” recruitment strategy to ensure high standards “departments need to know more about their existing workforces, shortages …problems’ a teacher’s own school results "It’s not the only quality, but it’s an essential quality.”	Orientations to standards (RI), knowing about workforce needs (MI) & the problems inherent in the profession (TI)

*Schools in some Australian states moved to online learning from the last week of March 2020 due to COVID restrictions

Article 10 could not be assessed for any particular orientation and Articles 2 and 3 are the same article published in two papers. This leaves 10 articles for discussion. Important to our work in this paper, only two articles (2/3 & 6) drew attention to the TPA, both written by the Director of the Queensland College of Teachers (the regulating authority that is responsible for registration for teachers in the state of Queensland). The TPA is neither mentioned by a journalist nor other stakeholders. We pick up on this point in the analysis of data set #2.

Our next observation concerns the orientations towards ITE that predominate in this suite of articles. All 10 articles had some orientation to RIs, reflecting the dominant view that quality ITE can only exist as a result of tight and conservative regulation. Nine of the 10 articles made direct reference to entry standards, some calling on entry standards to be raised and others appreciating that these benchmarks are not the sole determiner of quality teachers. Five of the articles mentioned LANTITE scores as being an important contributor to the discussion on preservice and graduate teacher quality. Four of the articles, two by journalists and two by other stakeholders, orientated only to RIs. Such an orientation provides a very narrow viewpoint about what counts as a quality ITE program. In the remaining six articles, RIs were combined with one or more of the other orientations. Five articles (2/3, 6, 8, 9 & 12) reflected orientations to TIs. Articles 6 and 7 reflected orientations to PIs, and Articles 8 and 12 reflected orientations to MIs. Only three Articles, 6 (other stakeholder), 8 (journalist) and 12 (journalist), drew attention to three of the identity options, indicating a more sophisticated understanding of important components of quality ITE program. There was no discernible pattern separating pre-COVID and COVID eras.

#### Analysis data set #2: social media

Given the dearth of public commentary on the implementation of the TPA in Australia in the legacy media, we were interested to see how this compared with social media posts. The 46 social media posts that data set #2 comprised were comments by preservice teachers in their final year of ITE regarding their final professional experience placement, the TPA or both. For a few, the only reference to the TPA was the inclusion of hashtags such as #gtpa. A number of posts supplemented a relevant hashtag with images that tied their comments to the TPA. Several posts included comments evaluating the TPA, for example as a hurdle or goal post, ‘the hardest thing I’ve ever done’, ‘the big bad TPA’, and ‘my baby’. Comments referred to the affective consequences of anticipating, executing and completing the TPA, for example, one post referred to ‘many rants and tears’. The emotions communicated in the posts ranged from apprehension to excitement, from despair to relief, pride and celebration. The posts were collegial in nature, offering encouragement and emotional support, and providing and requesting advice, ideas and resources. Some offered insights into their understanding of the content and possible expectations of the TPA. All the posts indicated a belief that efforts to complete the TPA both contributed to and demonstrated the development of teaching capabilities.

The PSTs perception of their development and demonstration of teaching quality is attested to in many of the posts, through the words but also by the inclusion of selfies that show the preservice and graduate teachers brimming with pride and jubilation. One selfie captured a mock celebration, a picture frame of the GTPA logo beside the score 5/5 held aloft like a trophy. Another selfie captured a celebratory portrait with an affirming comment about the GTPA displayed in a handheld light box. Both selfies required careful execution, and thus reflect the significance of this major capstone task that orientated to PIs. We also noted that the reach of these 46 posts seems to be limited to no more than a few dozen followers who appeared to be close contacts of the preservice or graduate teachers. Similar to data set #1 analysis, there was no discernible pattern separating pre-COVID and COVID eras.

## Discussions and conclusions

Returning to the overarching research question –What are the discourses driving the public commentary of AITSL’s Teaching Performance Assessment task in Australia and what are the inherent implications?—we note three key findings. The first finding is that the legacy media makes no clear distinction between the TPA with other ITE initiatives such as LANTITE. It could be that LANTITE, as a quantifiable and standardised assessment, is easier to understand and report. The second finding is that the TPA information shared by social media is from preservice teachers only, has a limited reach, and has no contributions from the ITE providers themselves. The third finding is that the social media commentaries about the TPA demonstrate that the preservice and graduate teachers recognise the weight of this major capstone assessment task. Findings one and two are new insights, and finding three aligns with research in Australia and elsewhere. Kriewaldt et al. (2021) completed a thematic data analysis of survey responses from Australian preservice teachers who reported that through completing the A*f*GT, they gained an awareness of important teaching capabilities that fostered professional growth. Chandler-Olcott and Fleming (2017) investigated stakeholder perspectives during the implementation of the edTPA in New York State. Their preservice participants indicated that it was a stressful process, with an important ‘gatekeeping’ role to the profession, which provided an impetus for them to develop several teaching capabilities.

Our data suggest, however, that for the period under scrutiny, those providing legacy media commentary on the broader commission of quality ITE had not yet become aware of the role of the TPA in quality assuring the outcomes of ITE. In earlier research work, the authors (Exley et al., 2022) found that the legacy media promoted the TPA as akin to a test. The research in hand demonstrates that legacy media comments about ITE orientated to RIs, with some attention to TIs and less attention to MIs and PIs. To date, the labour of public commentary about the TPA has been left to the preservice and graduate teachers who use social media platforms with a small audience reach, and with no discernible input from the ITE providers themselves. Given that teacher quality is often portrayed negatively in the media (see Pendergast et al., 2019), and social media platforms can erupt into fractured and unpleasant spaces, we are not entirely surprised to see the preservice and graduate teachers’ hesitation to proliferate in this space. We also think the dearth of social media commentary from ITE providers presents as an untapped opportunity.

Back in 2017, Rowe proffered that close regulation of ITE programmes would direct the focus to the means of surveillance, measurements and accountability itself, rather than the ‘end-goals of accountability’ (p. 12), that is on the quality of the preservice and graduate teacher. Instead, we would be interested to see if an approach to accountability that features capacity-building with a particular regard for the outcomes of TPAs had the potential to shift the discourses on graduate teacher quality.

Our data are limited to a particular timeframe and also limited to available data. Our data, however, suggest that at the time of data collection, in the Australian public sphere little was known and discussed about TPAs. One reason for this is the time it takes for formal programme accreditation and reaccreditation processes. The findings from participation in TPA benchmarking activities do not need to be published until AITSL accreditation stage two (AITSL, 2018, p. 7), which typically takes place approximately five years after a programme has achieved accreditation stage one. Most programmes around the nation were not yet at this stage during the timespan under investigation. As more ITE programmes head towards accreditation stage two, we are looking forward to the opportunities for highlighting the evidence of ITE programme impact and the TPA’s contribution as a capacity-building requirement.

Our analysis of the legacy and social media outputs identifies the potential for a range of stakeholders to contribute more visibly to the public discussion on the quality of outcomes and the impact of ITE programmes in Australia. We have yet to see what might be possible when ITE providers use social media platforms to more clearly demonstrate that graduate teachers are meeting the needs and expectations of employers, orientating to the rapidly changing social world, and enacting complex theories of personal, social and cognitive development.

## Data Availability

Data comprise the legacy media and social media posts. Publication details for the legacy media are included in the references. Details of the social media posts are available by contacting the authors.
